# *LncRNA-CR11538* Decoys Dif/Dorsal to Reduce Antimicrobial Peptide Products for Restoring *Drosophila* Toll Immunity Homeostasis

**DOI:** 10.3390/ijms221810117

**Published:** 2021-09-18

**Authors:** Hongjian Zhou, Shengjie Li, Shanshan Wu, Ping Jin, Fei Ma

**Affiliations:** 1 Laboratory for Comparative Genomics and Bioinformatics & Jiangsu Key Laboratory for Biodiversity and Biotechnology, College of Life Science, Nanjing Normal University, Nanjing 210046, China; 181201007@njnu.edu.cn (H.Z.); lishengjie@njxzc.edu.cn (S.L.); 201202066@njnu.edu.cn (S.W.); 08258@njnu.edu.cn (F.M.); 2 Jiangsu Provincial Key Construction Laboratory of Special Biomass Byproduct Resource Utilization, School of Food Science, Nanjing Xiaozhuang University, Nanjing 211171, China

**Keywords:** *lncRNA-CR11538*, *Drosophila melanogaster*, NF-κB transcription factor Dif/Dorsal, antimicrobial peptides, innate immunity, toll pathway

## Abstract

Avoiding excessive or insufficient immune responses and maintaining homeostasis are critical for animal survival. Although many positive or negative modulators involved in immune responses have been identified, little has been reported to date concerning whether the long non-coding RNA (lncRNA) can regulate *Drosophila* immunity response. In this study, we firstly discover that the overexpression of *lncRNA-CR11538* can inhibit the expressions of antimicrobial peptides *Drosomycin* (*Drs*) and *Metchnikowin* (*Mtk*) in vivo, thereby suppressing the Toll signaling pathway. Secondly, our results demonstrate that *lncRNA-CR11538* can interact with transcription factors Dif/Dorsal in the nucleus based on both subcellular localization and RIP analyses. Thirdly, our findings reveal that *lncRNA-CR11538* can decoy Dif/Dorsal away from the promoters of *Drs* and *Mtk* to repress their transcriptions by ChIP-qPCR and dual luciferase report experiments. Fourthly, the dynamic expression changes of *Drs*, *Dif*, *Dorsal* and *lncRNA-CR11538* in wild-type flies (*w^1118^*) at different time points after *M. luteus* stimulation disclose that *lncRNA-CR11538* can help *Drosophila* restore immune homeostasis in the later period of immune response. Overall, our study reveals a novel mechanism by which *lncRNA-CR11538* serves as a Dif/Dorsal decoy to downregulate antimicrobial peptide expressions for restoring *Drosophila* Toll immunity homeostasis, and provides a new insight into further studying the complex regulatory mechanism of animal innate immunity.

## 1. Introduction

The innate immune system can effectively protect animals from various bacteria, fungi and other pathogens [1,2,3]. The innate immunity signaling pathways, such as the Toll signaling pathway, are evolutionarily conserved from fruit flies (*Drosophila melanogaster*) to humans and *Drosophila* has emerged as a powerful model organism for studying bacterial infections [4,5]. The Toll signaling pathway goes through a series of signaling cascades to induce NF-κB transcription factor Dif/Dorsal into the nucleus to activate the expressions of antimicrobial peptides (AMPs) in response to Gram-positive bacteria or fungal invasion in *Drosophila* [5,6]. At present, studies have demonstrated that certain specific proteins, acting as positive or negative modulators, can prevent excessive or insufficient immune responses of *Drosophila* Toll pathway [7,8,9,10,11,12,13,14,15,16,17,18]. Additionally, although many non-coding RNAs, such as miRNAs, can negatively regulate innate immune response of Toll signaling pathway [19,20,21,22,23,24], little is known about the regulatory role of long non-coding RNAs (lncRNAs) in the *Drosophila* Toll signaling pathway, and further study is needed.

LncRNAs are a class of non-coding RNA with transcripts > 200 nucleotides and their regulatory mechanisms are complex and diverse [25,26]. LncRNAs can act as signals, decoys, guides and scaffolds to control gene expression at the epigenetic, transcriptional and post-transcriptional levels [25,27]. Especially, the nucleus-localized lncRNA can participate in regulating the binding of transcription factors to gene promoter regions at the transcription level or modulating epigenetic factors, which has been well studied in mammals [28]. For example, *lncRNA-NRON* inhibits the nuclear transport of dephosphorylated transcription factor nuclear factor activated T cells (NFAT) by interacting with importin-β superfamily members [29]. *LncRNA*-*CCND1* can repress *CCND1* transcription by recruiting TLS to the promoter of *CCND1* [30].

LncRNAs play critical roles in *Drosophila* embryo development [31,32,33,34,35,36], bristle formation [37,38], gonadal cell production [39,40], and neuromuscular junctions [41,42]. Several lncRNAs have been discovered to promote *Drosophila* Toll immune response. For example, *lncRNA-IBIN* was reported to enhance Toll pathway signaling, but was later discovered to be a putative peptide-encoding gene [43]. *VINR* has been reported to participate in the antiviral immunity of *Drosophila* and can regulate the Toll pathway [44]. Our previous work demonstrates that *lncRNA-CR46018* can promote *Drosophila* Toll pathway signaling [45]. However, whether and how lncRNA negatively regulate *Drosophila* Toll pathway remain unclear up to now.

In this study, we detected the role of the differentially expressed *lncRNA-CR11538* in *Drosophila* immune responses after *M. luteus* infection based on our previous work [46]. We constructed the CR11538-overexpressing flies and detected the expression levels of AMPs *Drs* and *Mtk* at 12 h after *M. luteus* infection by transcriptome sequencing as well as RT-qPCR. The subcellular localization assay and RIP experiments demonstrated that *lncRNA-CR11538* could interact with transcription factor Dif/Dorsal of the *Drosophila* Toll pathway. Both ChIP-qPCR and dual luciferase reporter assays demonstrated that *lncRNA-CR11538* can act as a decoy to sequester binding of Dif/Dorsal to the promoters of *Drs* and *Mtk* and inhibit the expressions of *Drs* and *Mtk*. Finally, the dynamic expression changes of *Drs*, *Dif*, *Dorsal*, *lncRNA-CR11538* in wild-type fruit flies after *M. luteus* infection indicated that the upregulated *lncRNA-CR11538* can help fruit flies restore immune homeostasis in the later of immune response. Taken together, we discovered a novel *lncRNA-CR11538* that can negatively regulate *Drosophila* Toll pathway via sequestering Dif/Dorsal away from the promoters of AMPs to suppress their transcriptions.

## 2. Results

### 2.1. The Overexpression of lncRNA-CR11538 Reduces AMP Productions of Toll Pathway

**Figure 1:**
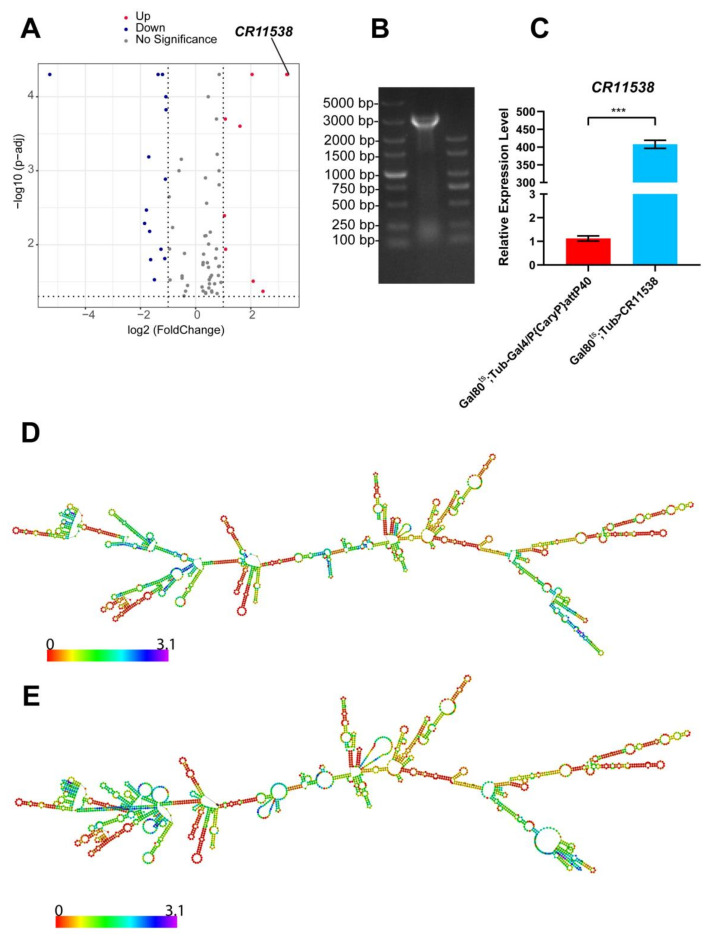
LncRNA-CR11538 is highly expressed after M. luteus stimulation. (**A**) Volcano map showing these differentially expressed lncRNAs in RNA-seq between PBS-treated and *M. luteus*-infected *w^1118^* flies. (**B**) PCR was performed using a forward primer for pUAST-attB and a reverse primer for *lncRNA-CR11538*. (**C**) The *lncRNA-CR11538* expression level was detected in the CR11538-overexpressing flies, normalized to their control levels. Minimum free energy method (**D**) and thermodynamic ensemble method (**E**) were used to predict its secondary structure on *RNAfold* Webserver. For all tests, *p* value < 0.05 was considered as statistically significant. *** *p* < 0.001 vs. the control groups.

In this study, we investigated the most significantly upregulated *lncRNA-CR11538* demonstrated by our previous work (Figure 1A) [46], and it was predicted to have no open reading frame by multiple bioinformatics tools. To explore whether *lncRNA-CR11538* can participate in the regulation of *Drosophila* immunity responses, we constructed the CR11538-overexpressing flies using the pUAST-attB plasmid method, and confirmed that *lncRNA-CR11538* was successfully constructed to the desired position in *Drosophila* genome (Figure 1B). To overexpress *Drosophila* *lncRNA-CR11538* in a specific period of time, these overexpressing *lncRNA-CR11538* transgenic flies were transiently driven by a temperature sensitive *Tub-Gal80^ts^; Tub-Gal4* flies were especially influenced; the expression level of *lncRNA-CR11538* was about 400 times higher than the control (Figure 1C). Additionally, the total length of *lncRNA-CR11538* is 2386 bp, and its secondary structure was predicted by applying the minimum free energy and thermodynamic ensemble method, respectively [47,48]. As shown in Figure 1D,E, the secondary structure of *lncRNA-CR11538* is very complex.

**Figure 2:**
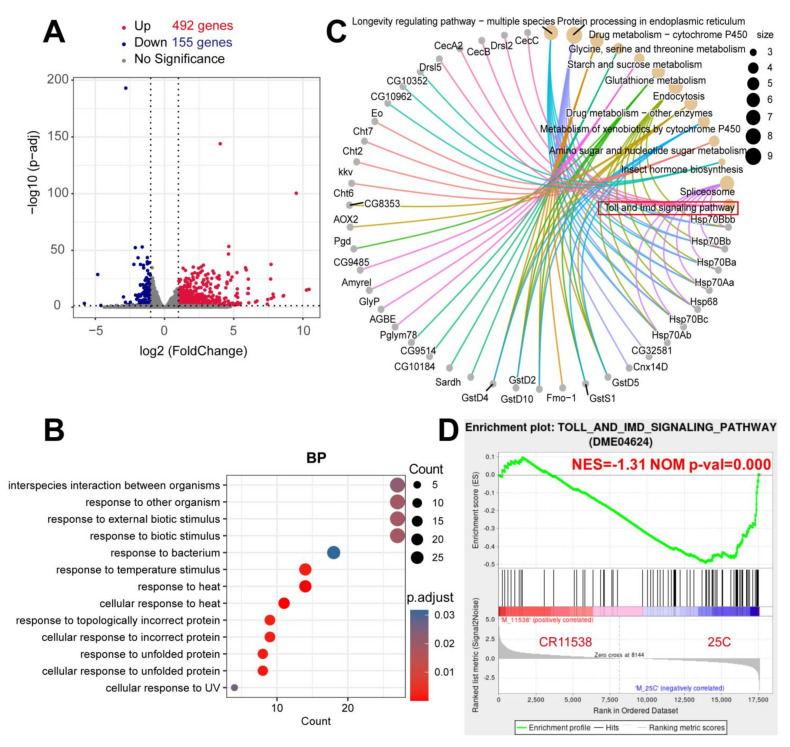
Enrichment analysis of DEGs in the CR11538-overexpressing flies after stimulation with *M. luteus*. (**A**) Volcano map showing DEGs among *Gal80^ts^; Tub> CR11538 and Gal80^ts^*; *Tub-Gal4/P{CaryP}attP40* after *M. luteus* challenge. Red: upregulated genes (>2-fold difference in relative expression and adjusted *p* < 0.05); blue: downregulated genes (<−2-fold difference in relative expression and adjusted *p* < 0.05). (**B**) Biological process enrichment analysis results were obtained using all DEGs. (**C**) Network diagram showing pathways and corresponding genes in KEGG pathway enriched with all DEGs. “size” represents the number of differentially expressed genes (DEGs) contained in each pathway. (**D**) GSEA analysis on the RNA-seq data between the *CR11538*-overexpressing flies and the control. The GSEA diagram is divided into three parts. The first part is the line graph of gene Enrichment Score. The second part uses lines to mark the genes under the gene set. The third part is the rank value distribution map of all genes.

We further performed transcriptome sequencing on the CR11538-overexpressing flies and the control flies at 12 h after infection with *M. luteus*. The transcriptome analysis results indicated that there are a total of 647 differentially expressed genes (DEGs) (|log2 (fold change)| > 1 and adjusted *p* value < 0.05) including 492 upregulated and 155 downregulated genes (Figure 2A). Gene ontology (GO) annotation and Kyoto encyclopedia of genes and genomes (KEGG) enrichment analysis revealed that these DEGs are significantly enriched in biological process annotation, the response to bacterial and other biological stimuli (Figure 2B), including the Toll signaling pathway (Figure 2C). Therefore, to detect the expression levels of genes of the Toll and Imd pathways in the CR11538-overexpressing flies, we performed gene set enrichment analysis (GSEA) analysis on the RNA-seq data and found that the overall gene expressions of the Toll and Imd pathways are downregulated with a normalized enrichment score of −1.31 and *p*-value = 0.000 (Figure 2D). These results suggest that *lncRNA-CR11538* could be involved in innate immune response to the invasion of *M. luteus*. Remarkably, these results were from the stimulation with *M. luteus*, so we mainly focused on the Toll pathway instead of the Imd pathway.

**Figure 3:**
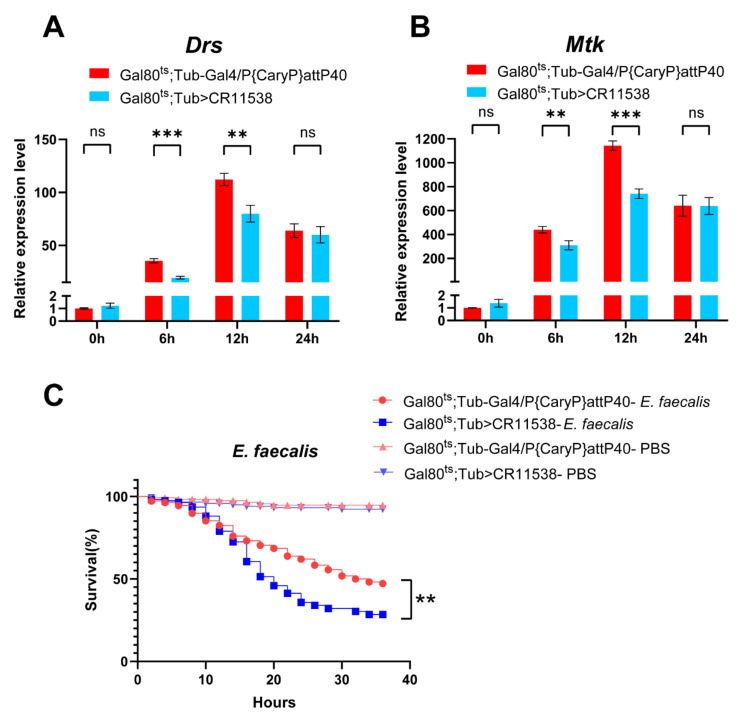
*lncRNA-CR11538* inhibits Toll signaling pathway in vivo. *Drs* (**A**) and *Mtk* (**B**) expression levels in the CR11538-overexpressing flies at different time points (0 h, 6 h, 12 h, 24 h) after *M. luteus* infection. (**C**) Changes in the survival rate of the CR11538-overexpressing flies and the control flies treated with PBS or *E. faecalis*. Measurements made after 36 h. *Gal80^ts^*; *Tub-Gal4/P{CaryP}attP40*-PBS (*n* = 115), *Gal80^ts^*; *Tub > CR11538*-PBS (*n* = 118), *Gal80^ts^*; *Tub-Gal4/P{CaryP}attP40*-*E. faecalis* (*n* = 108), *Gal80^ts^*; *Tub > CR11538*-*E. faecalis* (*n* = 109). For all tests, *p* value < 0.05 was considered as statistically significant. ** *p* < 0.01, *** *p* < 0.001 and ns, no significance vs. the control groups.

To verify the effect of *lncRNA-CR11538* on Toll signaling pathway in vivo, we examined the expression levels of *Drosomycin* (*Drs*) and *Metchnikowin* (*Mtk*), two signature AMPs of Toll pathway, at different time points in the CR11538-overexpressing flies after infection with *M. luteus*. Our results demonstrate that the expressions of *Drs* and *Mtk* are significantly downregulated in the CR11538-overexpressing flies compared to the control group at both 6 h and 12 h post infection, but no significant differences were seen at 24 h post infection (Figure 3A,B). Of note, to avoid the nonspecific or false positive results, we also constructed the dsRNA-mediated *lncRNA-CR11538* knockdown fly in *w^1118^* flies to test the expression levels of the antimicrobial peptides. Our findings demonstrated that the expressions of *Drs* and *Mtk* in the dsRNA-mediated *lncRNA-CR11538* knockdown flies are significantly increased compared with the controls at 6 h after infection with *M. luteus* (Figure S1C–E). Additionally, the survival rate of the CR11538-overexpressing flies after infection with Gram-positive lethal bacteria, *Enterococcus faecalis* (*E. faecalis*), is significantly lower than that of the control group (Figure 3C), but the survival rate of the files treated with PBS was not significantly affected (Figure 3C). Overall, our results implied that *lncRNA-CR11538* can inhibit the transcriptions of AMPs to negatively regulate *Drosophila* Toll signaling. 

### 2.2. The Interaction of lncRNA-CR11538 with Dif/Dorsal 

**Figure 4:**
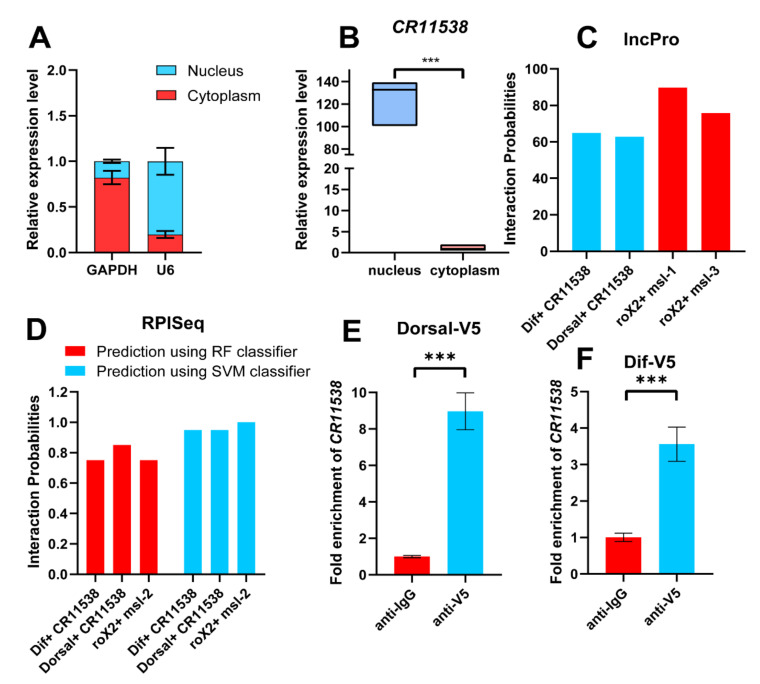
*LncRNA-CR11538* interacts with Dif/Dorsal. (**A**) *U6* and *GAPDH* are marker genes used to confirm partitioning between nucleus and cytoplasm. (**B**) *LncRNA-CR11538* expression in nucleus and cytoplasm detected by RT-qPCR. Predicted scores for interaction potential of *lncRNA-CR11538* with Dif or Dorsal via IncPro (**C**) and RPISeq (**D**) databases. *LncRNA-CR11538* fold enrichment measured by RIP-qPCR using anti-V5 antibody to immunoprecipitate overexpressed Dorsal-V5 (**E**) and Dif-V5 (**F**) in S2 cells. For all tests, *p* value < 0.05 was considered as statistically significant. *** *p* < 0.001 vs. the control groups.

To explore how *lncRNA-CR11538* negatively regulates the Toll pathway, we need to know the distribution of *lncRNA-CR11538* because lncRNA has different functions in the nucleus and the cytoplasm. The subcellular location showed that *lncRNA-CR11538* is mainly distributed in the nucleus by a nucleocytoplasmic separation experiment (Figure 4A,B). It is well-known that AMPs of the Toll signaling pathway are mainly transcribed by transcription factors Dif/Dorsal in the nucleus. This seemed to imply that *lncRNA-CR11538* could act as a Dif/Dorsal decoy to exert transcriptional repression effect on AMPs. Interestingly, *lncRNA-CR11538* and Dif/Dorsal were predicted to interact by the lncPro and RPISeq websites by taking the interaction between roX2 and msl as a positive control (Figure 4C,D). More importantly, we performed the RIP experiment and confirmed the interaction between *lncRNA-CR11538* and Dif/Dorsal using V5 antibody to immunoprecipitate Dorsal-V5 and Dif-V5 expressed in S2 cells (Figure 4E,F).

### 2.3. LncRNA-CR11538 Prevents Binding of Dif/Dorsal to AMPs Promoters

**Figure 5:**
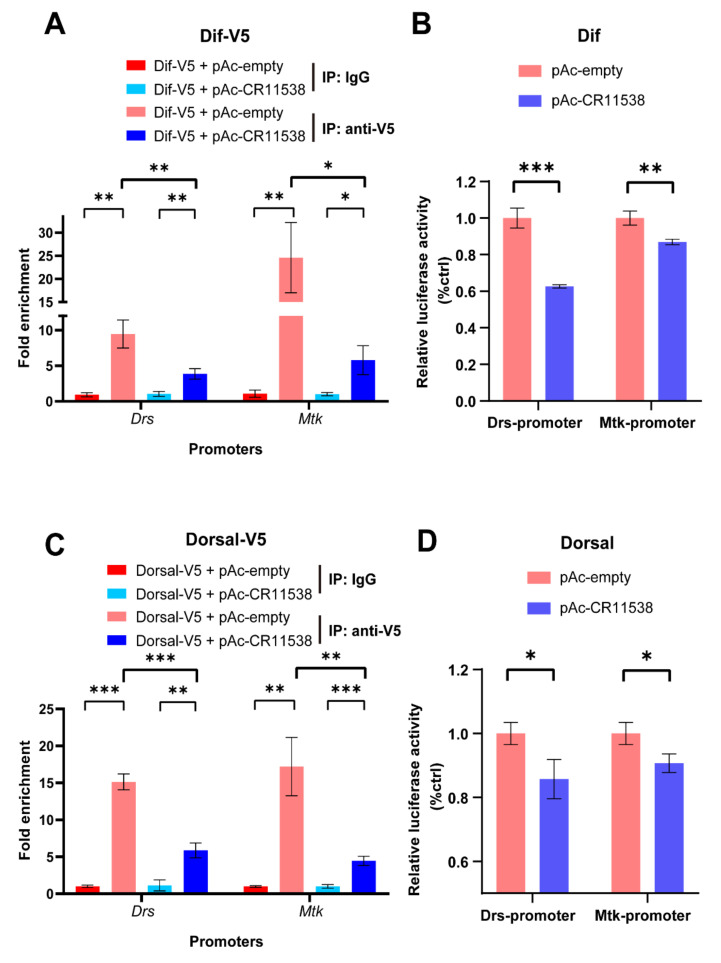
*LncRNA-CR11538* prevents Dif/Dorsal from binding to AMP promoters and inhibits their transcriptions. ChIP-qPCR was performed on *lncRNA-CR11538* overexpressing S2 cells with Dif-V5 (**A**) and Dorsal-V5 (**C**) overexpression normalized to control expression levels. The dual luciferase reporter assay detected transcriptional activity of Dif (**B**) and Dorsal (**D**) with or without *lncRNA-CR11538* overexpressing on *Drs* and *Mtk* promoters. For all tests, *p* value < 0.05 was considered as statistically significant. * *p* < 0.05; ** *p* < 0.01 and *** *p* < 0.001 vs. the control groups.

To reveal whether *lncRNA-CR11538* can serve as a decoy to prevent the binding of Dif/Dorsal to the promoters of AMPs, we co-expressed Dif/Dorsal-V5 and *lncRNA-CR11538* into S2 cells. The ChIP experiments showed that the highly expressed *lncRNA-CR11538* represses the binding of Dif/Dorsal-V5 to the promoters of *Drs* and *Mtk* (Figure 5A,C), whilst the over-expression of *lncRNA-CR11538* also leads to a significant decrease in the promoter activity of AMPs as detected by the dual luciferase reporter assay (Figure 5B,D). These results indicate that *lncRNA-CR11538* could function as a decoy to sequester binding of Dif/Dorsal to the promoters of AMPs, thereby inhibiting the promoter activity of AMPs to negatively regulate the Toll pathway in *Drosophila*.

### 2.4. LncRNA-CR11538 Facilitates Homeostasis Recovery in the Wild-Type Flies after Stimulation

**Figure 6:**
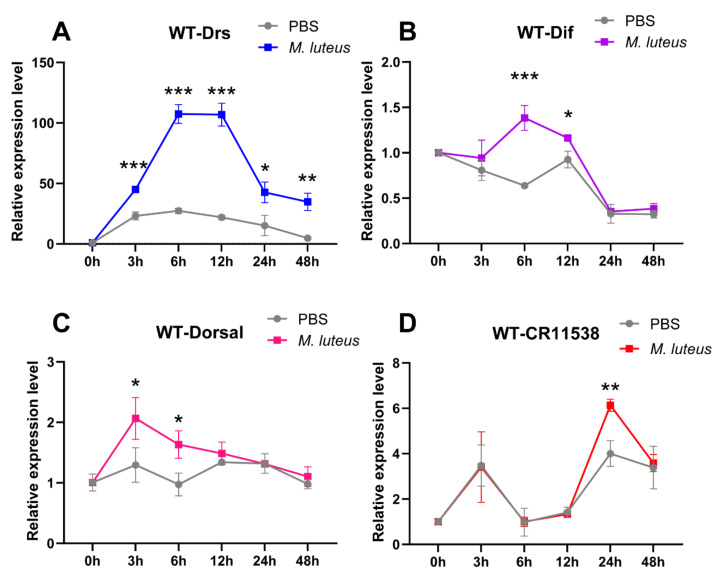
*LncRNA-CR11538* facilitates immune homeostasis recovery. The dynamic expression levels of *Drs* (**A**), *Dif* (**B**), *Dorsal* (**C**), *lncRNA-CR11538* (**D**) in the wild-type *Drosophila* infected with *M. luteus* were detected by qRT-PCR at 0 h, 3 h, 6 h, 12 h, 24 h and 48 h after stimulation. For all tests, *p* value < 0.05 was considered as statistically significant. * *p* < 0.05; ** *p* < 0.01 and *** *p* < 0.001 vs. the control groups.

To reveal the physiological role of *lncRNA-CR11538* in *Drosophila*, we examined the dynamical expression changes of *Drs*, *Dif*, *Dorsal*, and *lncRNA-CR11538* in wild-type flies (*w^1118^*) at 0 h, 3 h, 6 h, 12 h, 24 h, 48 h after *M. luteus* stimulation. Our results demonstrate the expression level of *Drs* is significantly upregulated at 3 h after *M. luteus* stimulation compared with the control flies and the peak occurs at 6–12 h after *M. luteus* stimulation, then gradually declined and almost returned to the original level at 24 and 48 h after *M. luteus* stimulation (Figure 6A). As expected, the NF-κB transcription factors *Dif* and *Dorsal* are significantly activated in the early stage of stimulation with *M. luteus* (*Dif* at 6–12 h, *Dorsal* at 3–6 h) and have no significant difference at other time points compared with the control (Figure 6B,C). Remarkably, the *lncRNA-CR11538* is highly expressed in the later stage of stimulation with *M. luteus* (24 h), but no significant change occurs at other time points (Figure 6D). Taking all of the above results together, it seems reasonable that in the early stage of stimulation with *M. luteus*, the *Drosophila* NF-κB transcription factors *Dif* and *Dorsal* are upregulated and then promote the transcription of AMPs via binging to their promoters. Nonetheless, to prevent excessive immune response in the later stage of stimulation with *M. luteus*, *lncRNA-CR11538* is increased and acts as a Dif/Dorsal decoy to lead them away from the AMP promoters and restore immune homeostasis.

## 3. Discussion

Insects and mammals share conserved innate immunity pathways, such as the Toll signaling pathway in response to the invasion of Gram-positive bacteria and fungi [5,49]. Therefore, studying further *Drosophila* innate immune regulators can deepen our understanding of mammalian immune regulation. Although many positive or negative protein modulators have been discovered to participate in Toll pathway immune responses, to date only a few lncRNAs (e.g., *CR46018* and *VINR*) have been found to positively regulate the *Drosophila* Toll pathway. It remains unclear how lncRNAs negatively regulate the *Drosophila* innate immune system to eliminate excessive immune responses [43,44,45]. In our study, we were delighted to find that *lncRNA-CR11538* can decoy Dif/Dorsal away from AMP promoters and inhibit their transcriptions, thereby negatively regulating Toll pathway signaling to prevent excessive immune activation in *Drosophila* with *M. luteus* infection.

Transcriptome sequencing is a very important tool for identifying functional lncRNAs. At present, most studies have used the transcriptome sequencing to identify new regulatory lncRNAs and reveal their diversified functions [50,51]. In our work, we used transcriptome sequencing to demonstrate that *lncRNA-CR11538* can inhibit the *Drosophila* Toll pathway (Figure 2), and can be involved in regulating the Toll pathway, the longevity pathway and other metabolic detoxification pathways by KEGG enrichment analysis and GSEA analysis (Figure 2C,D). In addition, the results from the transcriptome sequencing indicate that AMPs of the Imd pathway are also differentially expressed in CR11538-overexpressing flies after *M. luteus* stimulation. Herein, considering the results are from *M. luteus* stimulation, it is reasonable to argue that the question of whether *lncRNA-CR11538* can regulate Imd pathway needs further study.

The functions of lncRNAs are diverse [27]. lncRNAs have different roles in the nucleus and outside the nucleus. In the nucleus, lncRNAs can act as signals, decoys, guides and scaffolds to be involved in the regulation of gene transcription and epigenetics, thereby performing diverse functions. [25,28]. Interestingly, studies have indicated that lncRNAs can interact with NF-κB transcriptional factor in mammals [52,53]. These NF-κB transcriptional factors are evolutionarily conserved between *Drosophila* and mammals, which seems to imply that *lncRNA-CR11538* can also interact with the NF-κB Dif/Dorsal. As expected, our results demonstrate that *lncRNA-CR11538* can act as a decoy to trap Dif/Dorsal away from the promoters of *Drs* and *Mtk* in the nucleus (Figure 5), thereby inhibiting the production of AMPs to further repress the *Drosophila* Toll pathway.

**Figure 7:**
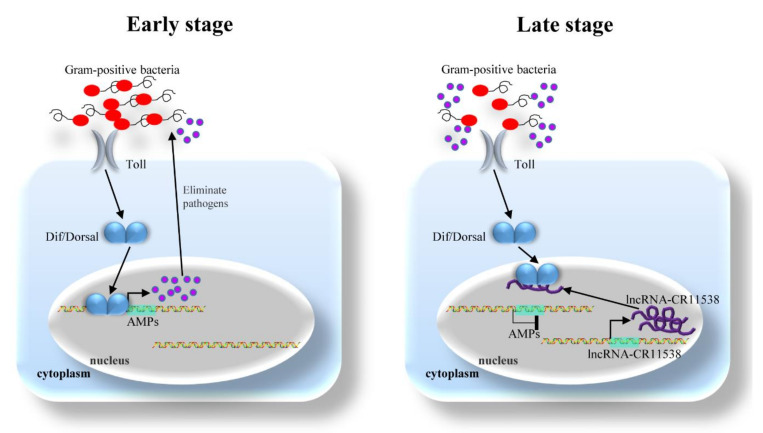
Schematic illustration of the mechanism by which *lncRNA-CR11538* facilitates immune homeostasis recovery. Left diagram: In the early stage of immune response, Gram-positive bacteria activate Toll signaling pathway and promote Dif/Dorsal entering the nucleus to enhance AMP transcriptions, thereby eliminating pathogens. Right diagram: In the late stage of immune response, redundant AMPs increase the expression of *lncRNA-CR11538*, which acts as a Dif/Dorsal decoy to sequester them away from AMP promoters and inhibits the transcriptions of AMPs to restore immune homeostasis.

Taken together, we found a new *lncRNA-CR11538* that negatively regulates the Toll signaling pathway and contributes to immune homeostasis recovery in *Drosophila*. In brief, *lncRNA-CR11538* is highly expressed in *Drosophila* in the later stage of stimulation with *M. luteus* (24 h), and acts as a Dif/Dorsal decoy in the nucleus to lead Dif/Dorsal away from the promoters of AMPs, thereby inhibiting the expression of AMPs to facilitate immune homeostasis recovery in the later stage of *M. luteus* stimulation (Figure 7). All in all, our study deepened our understanding on the roles played by lncRNA in regulating the *Drosophila* Toll pathway, and provided new insights into the study of the innate immune system of insects and mammals.

## 4. Materials and Methods

### 4.1. Fly breeding, Strain and Model Construction

*Drosophila* were grown in a medium composed of corn meal/agar/yeast, the culture temperature was maintained at 24 ± 1 °C, and the light/dark cycle was 12 h. The fly stocks *w^1118^* (#3605), *Tub-Gal80^ts^; TM2/TM6B* (#7019), *Tub-Gal4/TM3, Sb1, Ser1* (#5138) were obtained from the Bloomington Drosophila Stock Center (Bloomington, IN, USA). To generate CR11538-overexpressing flies, the full-length *lncRNA-CR11538* was constructed into the pUAST-attB vector using primers listed in Supplementary Table S1. The constructed plasmid was injected into embryos of attp2# (25C6): *y* [1] M{vas-int.Dm}ZH-2A w[*]; P{CaryP}attP40. In order to minimize the effect of *lncRNA-CR11538* on the development of *Drosophila*, the transgenic *CR11538* flies were crossed with temperature sensitive *Tub-Gal80^ts^; Tub-Gal4* flies and cultured at 18 °C, and transiently overexpressed *lncRNA-CR11538* once transferred to an incubator at 29 °C for 24 h for the following experiments.

### 4.2. Sepsis and Survival Experiments

Adult male flies were collected for 3 to 4 days. The control flies and CR11538-overexpressing flies were infected with *M. luteus* using a Nanoject instrument (Nanoliter 2010; WPI, Sarasota, FL, USA). A glass capillary filled with *M. luteus* suspension was inserted into the thorax of each fly and the insects were collected at the required detection times for the subsequent experiments. Postinfection survival rates indicate immune response deficiencies [54]. The 36 h survival rate was determined after infection of ≥ 100 flies/group with *Enterococcus faecalis* (*E. faecalis*) concentrate.

### 4.3. Genomic DNA Extraction and lncRNA Identification in Drosophila

Ten anesthetized flies were placed in a 1.5 mL centrifuge tube containing 100 μL digestion buffer (100 mM NaCl, 10 mM Tris-Cl [pH 8.0], 25 mM EDTA [pH 8.0], and 0.5% (*w*/*v*) SDS). The flies were pulverized and digestion buffer was added to make up the volume to 500 μL. The suspension was incubated in 65 °C water bath for 4 h and shaken once every 30 min to promote sample digestion. The centrifuge tube was removed and 400 μL phenol and 400 μL chloroform:isoamyl alcohol (24:1 *v*/*v*) were added to it. The tube was sealed, inverted, and vigorously mixed. The suspension was allowed to stand 5 min and centrifuged at 12,000× *g* for 10 min. The supernatant was decanted into an empty dry centrifuge tube. Then, 400 μL isopropanol was added and the suspension was gently mixed for 3 min and allowed to stand for 10 min. The samples were centrifuged at 14,000× *g* for 10 min and the white flakes adhered to the bottom of the tube. The precipitate was washed with 500 μL of 70% (*v*/*v*) ethanol, centrifuged. Discarded the ethanol, air-dried the samples. The precipitate was dissolved in 200 μL TE buffer. PCR was then performed using the forward primer of pUAST-attB and the reverse primer of *lncRNA-CR11538* (listed in Supplementary Table S1) to localize *lncRNA-CR11538* insertion into the *Drosophila* genome.

### 4.4. RNA Extraction and RT-qPCR

Total RNA was isolated from treated adult flies with RNA isolator total RNA extraction reagent (Vazyme Biotech Co. Ltd., Nanjing, Jiangsu, China). For RT-PCR, cDNA was prepared with a first-strand cDNA synthesis kit (Vazyme Biotech Co. Ltd., Nanjing, Jiangsu, China). The qPCR was performed in an ABI StepOne Plus real-time PCR system (Applied Biosystems, Foster City, CA, USA) with AceQ SYBR Green Master Mix (Vazyme Biotech Co. Ltd., Nanjing, Jiangsu, China). The mRNA expression levels were normalized to the *rp49* control. All experiments were conducted in triplicate. The relative 2^−^^△△Ct^ method was used for data analysis [55]. All primers used in this analysis are listed in Supplementary Table S2. All qPCR data are reported as means ± SEM.

### 4.5. RNA-Sequencing and Enrichment Analyses

RNA integrity was assessed with the RNA Nano 6000 assay kit for the Bioanalyzer 2100 system (Agilent Technologies, Santa Clara, CA, USA). PCR was performed with Phusion high-fidelity DNA polymerase, universal PCR primers, and index (X) primer. The PCR products were purified in the AMPure XP system (Beckman Coulter, Beverly, MA, USA) and library quality was assessed in the Agilent Bioanalyzer 2100 system (Agilent Technologies, Santa Clara, CA, USA). Index-coded samples were clustered on a cBot cluster generation system using a TruSeq PE cluster kit v3-cBot-HS (Illumina, San Diego, CA, USA) according to the manufacturer’s instructions. The reference genome index was built and paired-end clean reads were aligned to the reference genome with Hisat2 v2.0.5. FeatureCounts v. 1.5.0-p3 enumerated the reads mapped to each gene. Differential expression analysis was performed in the DESeq2 package of R v. 1.20.0 (R Core Team, Vienna, Austria). Genes with adjusted *p* < 0.05 and relative difference in expression level > 2 fold or <−2-fold were deemed differentially expressed. Gene ontology (GO) and Kyoto Encyclopedia of Genes and Genomes (KEGG) enrichment analyses were performed on the DEGs using the clusterProfiler package in R 4.0.3 (R Core Team, Vienna, Austria). Gene set enrichment analysis (GSEA) was used to determine whether a predefined gene set significantly differs between two biological states [56]. The local version of the GSEA tool (http://www.broadinstitute.org/gsea/index.jsp (18 August 2021)) was used for the GSEA and the KEGG datasets were used independently.

### 4.6. Subcellular Fractionation

RNA isolation was performed in accordance with the protocols of Rockland Immunochemicals [57]. In brief, 1 × 10^7^ S2 cells were resuspended in five volumes lysis buffer (10 mM HEPES, 60 mM KCl, 1 mM EDTA, 0.075% (*v*/*v*) NP-40, 1 mM DTT, and 1 mM PMSF adjusted to pH 7.6). The samples were then centrifuged at 1000 rpm for 3 min to separate the nuclear and cytoplasmic fractions. The supernatants were transferred to fresh tubes and the mixtures were aspirated with filter cartridges to isolate cytoplasmic RNA. The pellets were washed with ice-cold cell fractionation buffer and the nuclear RNA was isolated with TRIzol reagent (Invitrogen, Carlsbad, CA, USA). The RNA was then subjected to RT-qPCR analysis. The expression levels of the target genes in the individual fractions were normalized to their input expression levels.

### 4.7. Cell culture and Transfection

*Drosophila* S2 cells were maintained at 28 °C in SFX insect medium (HyClone Laboratories, Logan, UT, USA) supplemented with 10% (*v*/*v*) fetal bovine serum (FBS), 100 U/mL penicillin, and 100 μg/mL streptomycin (Invitrogen, Carlsbad, CA, USA). Full-length 2386bp *lncRNA-CR11538* was obtained by PCR with primers and integrated into the pAc5.1 plasmid. S2 cells were transiently transfected with 500 μL transfection complex in 6 mm plates (Corning, Corning, NY, USA) or with 50 μL transfection complex in 24-well plates (Corning, Corning, NY, USA) using X-treme gene HP transfection reagent (Roche Diagnostics, Basel, Switzerland) according to the manufacturer’s instructions.

### 4.8. Prediction of Interaction between RNA and Protein

The RPISeq RNA/protein interaction prediction tool (http://pridb.gdcb.iastate.edu/RPISeq/ (18 August 2021)) [58] and lncPro (http://bioinfo.bjmu.edu.cn/lncpro/# (18 August 2021)) were used to predict the interaction potential between lncRNA-CR11538 and Dif/Dorsal. Known interaction between roX2 and msl was the positive control.

### 4.9. RNA-Immunoprecipitation (RIP)

In total, 3 × 10^7^ transfected cells were dissolved in RIPA buffer (50 mM Tris-HCl [pH 7.4], 150 mM NaCl, 10 mM EDTA, 1% (*w*/*v*) NP-40, and 1 mM PMSF), RNase inhibitor (Thermo Fisher Scientific, Waltham, MA, USA) and protease inhibitor cocktail (Roche Diagnostics, Basel, Switzerland) on ice for 30 min. After centrifugation at 13,000× *g*, 4 °C for 15 min, the supernatants were pre-cleared with protein A agarose (Invitrogen, Carlsbad, CA, USA) at 4 °C for 1 h. Anti-V5-labeled or control IgG antibodies (ABclonal Biotechnology Co. Ltd., Wuhan, Hubei, China) were added to the pre-cleared supernatants and the samples were incubated at 4 °C for 4 h. Protein A agarose was then added and allowed to bind for 1~3 h. The conjugated beads were washed five times with RIPA buffer for 5 min each time. The remaining complexes were eluted with elution buffer (100 mM Tris [pH 8.0], 10 mM EDTA, and 1% (*w*/*v*) SDS). The samples were treated with protease K and the RNA was extracted with phenol/chloroform. The purified RNA was analyzed by RT-qPCR.

### 4.10. Transcription Factor (TF) Binding Site Prediction

The AMP promoter sequences were obtained from flybase (http://flybase.org/ (18 August 2021)). The Dif and Dorsal motifs were obtained from the literature [59]. All promoter sequences and motifs were submitted to the Multiple EM for Motif Elicitation database (MEME; https://meme-suite.org/meme/tools/meme (18 August 2021)). 

### 4.11. Chromatin Immunoprecipitation (ChIP)

ChIP was performed as previously described [60]. Transfected S2 cells were cross-linked with 1% (*v*/*v*) formaldehyde for 10 min. They were then lysed with cell and nuclear lysis buffers. Clarified lysates were then sonicated. The chromatin was sheared into 200–800-bp fragments and used in ChIP incubation with Dynabeads protein G (Thermo Fisher Scientific, Waltham, MA, USA) coated either with anti-V5 antibody (ABclonal Biotechnology Co. Ltd., Wuhan, Hubei, China) or anti-mouse IgG antibody on a rotating platform at 4 °C overnight. After repeated washings on a magnetic rack (Thermo Fisher Scientific, Waltham, MA, USA), the V5-bound genomic DNA was eluted from the Dynabeads and the cross-links were reversed at 65 °C overnight. The DNA fragments were purified with an AxyPrep PCR cleanup kit (Axygen Scientific, Union City, CA, USA). The qPCR analysis was performed using DNA from the Input and ChIP experiments and the primers listed in Supplementary Table S3. At least three independent experiments were conducted on the AMPs promoters. 

### 4.12. Dual Luciferase Reporter Assay

S2 cells were transfected with mixed transfection complex containing pIEx4-V5-Dif or pIEx4-V5-Dorsal, pGL3-Drs-promoter or pGL3-Mtk-promoter, pAc-empty or pAc-CR11538, and Renilla luciferase plasmid (pRL). Promega pRL (Promega, Madison, WI, USA) was used to normalize transfection efficiency. Luciferase activity was measured in a dual-luciferase reporter assay system (Promega, Madison, WI, USA) according to the manufacturer’s instructions.

### 4.13. Data Processing and Statistical Analysis

Experimental data were collected from three independent biological replicates and were presented as means ± SEM. Significant differences between values under different experimental conditions were subjected to two-tailed Student’s *t*-tests. Statistical fly survival analysis was performed using log-rank (Mantel–Cox) tests. Graphs were plotted with GraphPad Prism v. 8.3 (GraphPad Software, La Jolla, CA, USA) and RStudio (R Core Team, Vienna, Austria). *p* < 0.05 was considered statistically significant. * *p* < 0.05; ** *p* < 0.01; *** *p* < 0.001; ns, not significantly different from the control.

## Data Availability

The transcriptome analysis (RNA sequencing) datasets are available through the following links: The first transcriptome analysis (*M. luteus* induced genes compared to controls): https://www.ncbi.nlm.nih.gov/geo/query/acc.cgi?acc=GSE173733 (18 August 2021). The second transcriptome analysis (effect of *lncRNA-CR11538* overexpression and infection to gene expression on the whole transcriptome scale): https://www.ncbi.nlm.nih.gov/geo/query/acc.cgi?acc=GSE181870 (18 August 2021).

## Supplementary Materials

The following are available online at https://www.mdpi.com/article/10.3390/ijms221810117/s1. Figure S1: The expression levels of *IM1* (A), *IM2* (B), in control flies and the CR11538-overexpressing flies were measured at 6h after *M. luteus* infection. The expression levels of *lncRNA-CR11538* (C), *Drs*(D), *Mtk*(E) in control flies and the dsRNA-mediated lncRNA-CR11538 knockdown flies were measured at 6h after *M. luteus* infection. Figure S2: The dynamic expression levels of *lncRNA-CR46018* (A), *IBIN*(B), *VINR* (C) in the wild-type *Drosophila* infected with *M. luteus* were detected by qRT-PCR at 0 h, 3 h, 6 h, 12 h, 24 h and 48 h after stimulation. Table S2: Primers used for quantitative RT-PCR. Table S3: Primers used for ChIP-qPCR. Table S4: The differentially expressed genes (DEGs) in the transcriptome analysis results of CR11538-overexpressing flies and the control flies at 12 h after infection with *M. luteus*.
