# Supplementary material for: LncRNA-CR11538 Decoys Dif/Dorsal to Reduce Antimicrobial Peptide Products for Restoring Drosophila Toll Immunity Homeostasis
Source: Int J Mol Sci. 2021 Sep 18;22(18):10117. doi: 10.3390/ijms221810117 (PMC8468853; doi:10.3390/ijms221810117)
Supplement: Supplementary file 1 [file ijms-22-10117-s001.zip › Supplementary Table S2.pdf]

Supplementary Table S2. Primers used for quantitative RT-PCR :

Name	Primer sequence(5' – 3')
rp49-qF	GACGCTTCAAGGGACAGTATCTG
rp49-qR	AAACGCGGTTCTGCATGAG
Drs-qF	GTTCGCCCTCTTCGCTGTCC
Drs-qR	CCACTGGAGCGTCCCTCCTC
Mtk-qF	CGTCGCCCTTCAATCCTA
Mtk-qR	CGACATCAGCAGTGTGAATTT
GAPDH-qF	TAAATTCGACTCGACTCACGGT
GAPDH-qR	CTCCACCACATACTCGGCTC
CR11538-qF	GCCATACCAAGATCAATCCTACA
CR11538-qR	GCTTCACCTAACCACCAGTT
U6-qF	CTTCGGCAGAACATATACTAA
U6-qR	ATTTTGCGTGTCATCCTT
Dorsal-qF	ATCCGTGTGGATCCGTTTAA
Dorsal-qR	AATCGCACCGAATTCAGATC
Dif-qF	ATGTTTGAGGAGGCTTTCGG
Dif-qR	GAACCGGCGGTGCGACCCTCGC
