# Supplementary material for: LncRNA-CR11538 Decoys Dif/Dorsal to Reduce Antimicrobial Peptide Products for Restoring Drosophila Toll Immunity Homeostasis
Source: Int J Mol Sci. 2021 Sep 18;22(18):10117. doi: 10.3390/ijms221810117 (PMC8468853; doi:10.3390/ijms221810117)
Supplement: Supplementary file 1 [file ijms-22-10117-s001.zip › Supplementary Table S3.pdf]

Supplementary Table S3. Primers used for ChIP-qPCR:

Name	Primer sequence(5’ – 3’)
ChIP-Mtk-pro-qF	GTTTTCGTGGGAGGTGGAGA
ChIP-Mtk-pro-qR	GAATGAATGACGGGCAAGTGT
ChIP-Drs-pro-qF	AATGCTTTTCGCTTACGCTTTTCG
ChIP-Drs-pro-qR	GACTTGTGGCTTGGGAACTTCG
