# Supplementary material for: LncRNA-CR11538 Decoys Dif/Dorsal to Reduce Antimicrobial Peptide Products for Restoring Drosophila Toll Immunity Homeostasis
Source: Int J Mol Sci. 2021 Sep 18;22(18):10117. doi: 10.3390/ijms221810117 (PMC8468853; doi:10.3390/ijms221810117)
Supplement: Supplementary file 1 [file ijms-22-10117-s001.zip › ijms-1366115-Figure S1 and Figure S2.pdf]

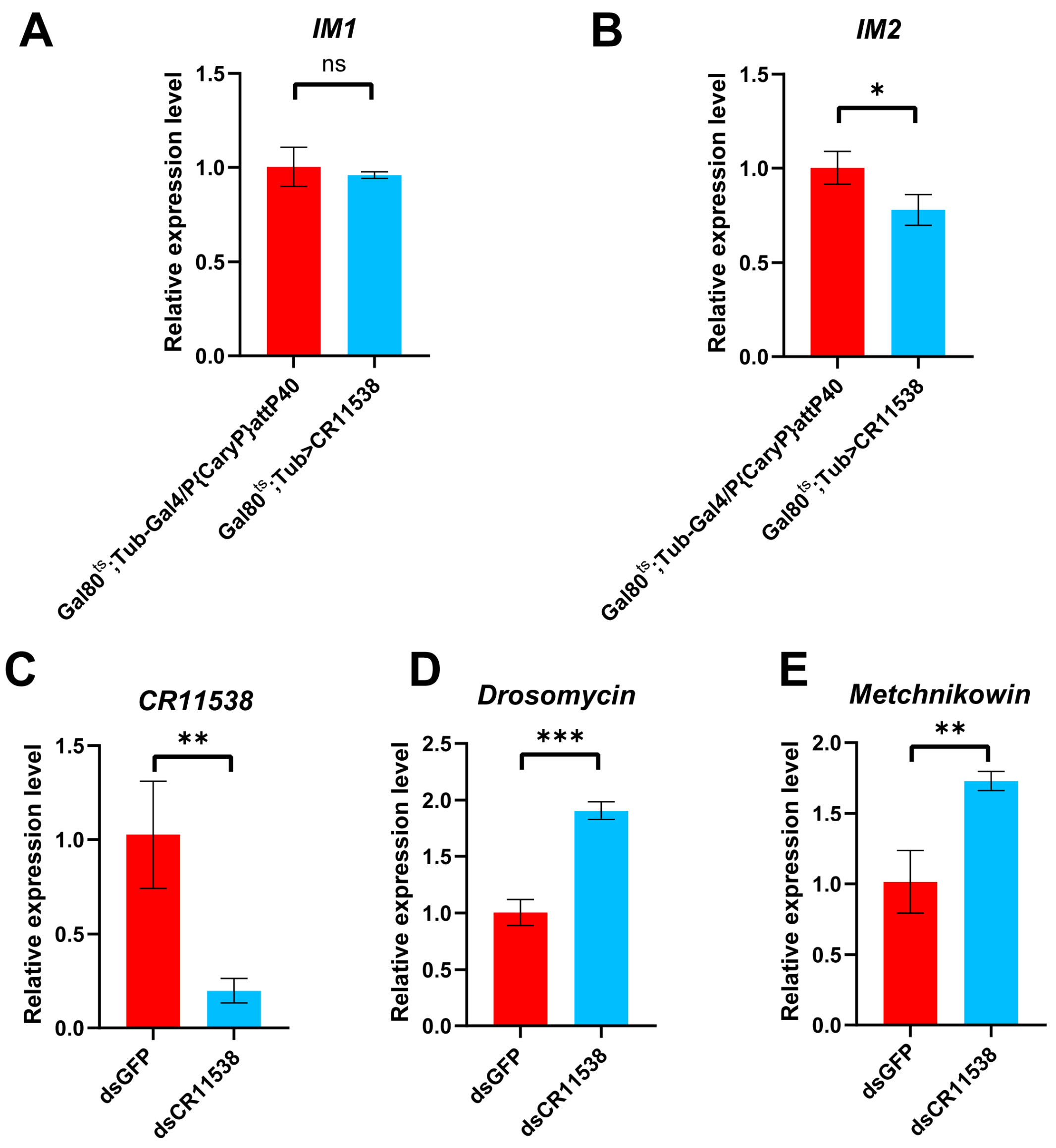

Figure S1: Figure S1: The expression levels of *IM1* (A), *IM2* (B), in control flies and the CR11538-overexpressing flies were measured at 6h after *M. luteus* infection. The expression levels of *lncRNA-CR11538* (C), *Drs*(D), *Mtk*(E) in control flies and the dsRNA-mediated lncRNA-CR11538 knockdown flies were measured at 6h after *M. luteus* infection. For all tests, *P* value < 0.05 was considered as statistically significant. * *P* < 0.05; ** *P* < 0.01, *** *P* < 0.001 and ns, no significance vs. the control groups

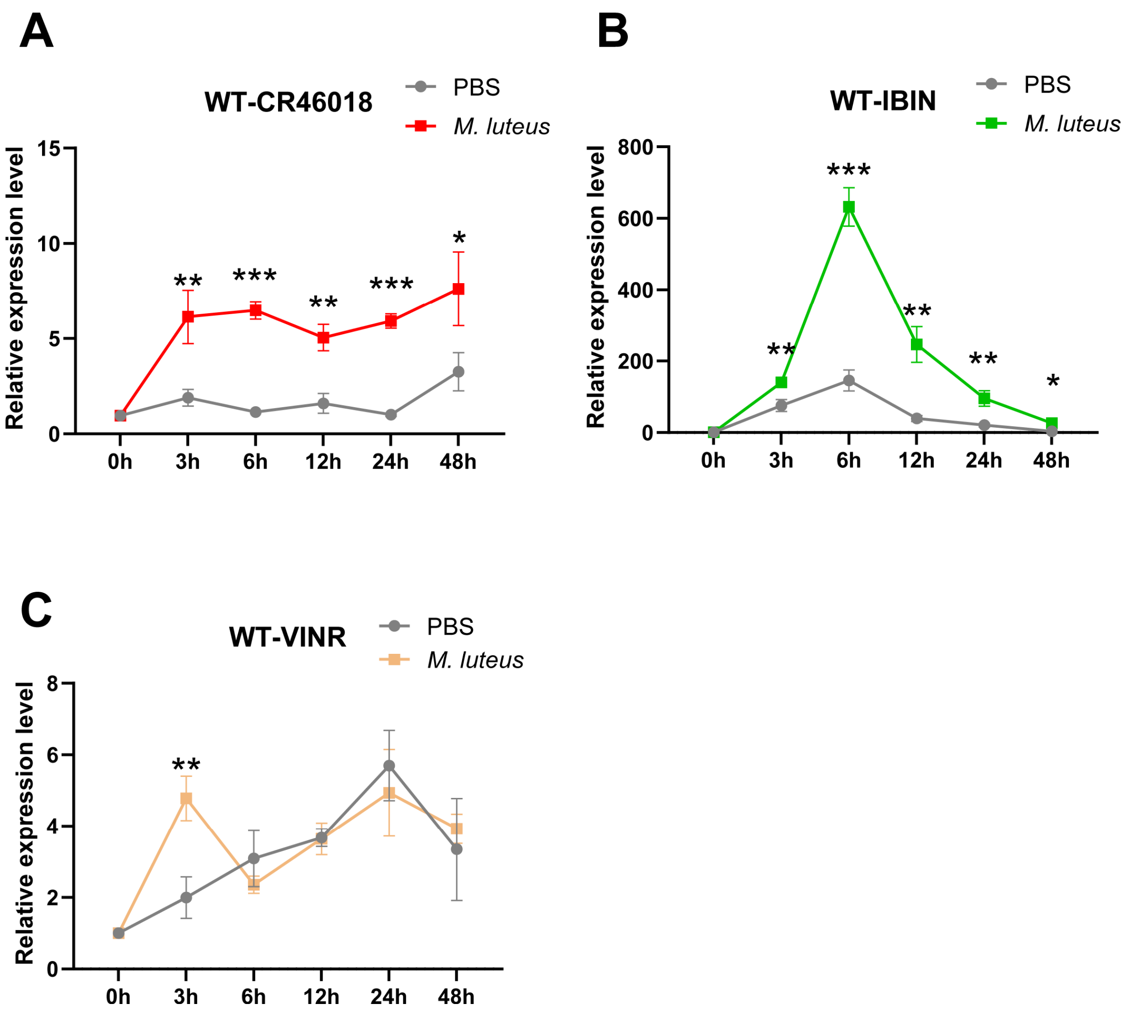

Figure S2: The dynamic expression levels of *lncRNA-CR46018* (**A**), *IBIN*(**B**), *VINR* (**C**) in the wild-type *Drosophila* infected with *M. luteus* were detected by qRT-PCR at 0 h, 3 h, 6 h, 12 h, 24 h and 48 h after stimulation. For all tests, *P* value < 0.05 was considered as statistically significant. * $P < 0.05$; ** $P < 0.01$ and *** $P < 0.001$ vs. the control groups.
