# Supplementary material for: LncRNA-CR11538 Decoys Dif/Dorsal to Reduce Antimicrobial Peptide Products for Restoring Drosophila Toll Immunity Homeostasis
Source: Int J Mol Sci. 2021 Sep 18;22(18):10117. doi: 10.3390/ijms221810117 (PMC8468853; doi:10.3390/ijms221810117)
Supplement: Supplementary file 1 [file ijms-22-10117-s001.zip › Supplementary Table S1.pdf]

Supplementary Table S1. Primers used for transgene vector construction:

Name	Primer sequence(5' – 3')
pUAST-attB-CR11538-F	CGGAATTCATGGAAGATCTTCACGTCGA
pUAST-attB-CR11538-R	CGCTCGAGAACTGGATCTTCCAGCTGCT
pAc-CR11538-F	CGGAATTCATGGAAGATCTTCACGTCGA
pAc-CR11538-R	CGCTCGAGAACTGGATCTTCCAGCTGCT
pUAST-attB-F	GTTCGGAGTGATTAGCGT
pUAST-attB-R	TGTTGAGAGTCAGCAGTAGC
